# The Neural Correlates of Face-Voice-Integration in Social Anxiety Disorder

**DOI:** 10.3389/fpsyt.2020.00657

**Published:** 2020-07-15

**Authors:** Benjamin Kreifelts, Thomas Ethofer, Ariane Wiegand, Carolin Brück, Sarah Wächter, Michael Erb, Martin Lotze, Dirk Wildgruber

**Affiliations:** ^1^Department of Psychiatry and Psychotherapy, University of Tübingen, Tübingen, Germany; ^2^Department for Biomedical Magnetic Resonance, University of Tübingen, Tübingen, Germany; ^3^Functional Imaging Group, Department for Diagnostic Radiology and Neuroradiology, University of Greifswald, Greifswald, Germany

**Keywords:** social anxiety disorder, superior temporal sulcus, audiovisual integration, functional magnetic resonance imaging, psycho-physiological interaction, temporal voice area

## Abstract

Faces and voices are very important sources of threat in social anxiety disorder (SAD), a common psychiatric disorder where core elements are fears of social exclusion and negative evaluation. Previous research in social anxiety evidenced increased cerebral responses to negative facial or vocal expressions and also generally increased hemodynamic responses to voices and faces. But it is unclear if also the cerebral process of face-voice-integration is altered in SAD. Applying functional magnetic resonance imaging, we investigated the correlates of the audiovisual integration of dynamic faces and voices in SAD as compared to healthy individuals. In the bilateral midsections of the superior temporal sulcus (STS) increased integration effects in SAD were observed driven by greater activation increases during audiovisual stimulation as compared to auditory stimulation. This effect was accompanied by increased functional connectivity with the visual association cortex and a more anterior position of the individual integration maxima along the STS in SAD. These findings demonstrate that the audiovisual integration of facial and vocal cues in SAD is not only systematically altered with regard to intensity and connectivity but also the individual location of the integration areas within the STS. These combined findings offer a novel perspective on the neuronal representation of social signal processing in individuals suffering from SAD.

## Introduction

Social anxiety (SA) can be conceptualized as maladaptive evolutionary mechanism which developed parallel to a social hierarchy which was no longer based on physical dominance but rather social skills relying on nonverbal and later verbal communication signals (1, 2). The clinical manifestation of SA, termed social anxiety disorder (SAD), is a common psychiatric disorder (3) and forms the upper end of an SA severity spectrum spanning the general population. The central elements of SA relate to exclusion, humiliation, and negative social evaluation in general. The threat thereof is typically communicated not only *via* facial but also vocal signals in multimodal face-to-face communication. Consistently, one of the key features in SAD are cognitive biases towards socially threatening signals in faces and voices (4). Correspondingly, increased responses to such signals were observed in the cerebral emotion and salience processing networks [i.e., the amygdala, insula, mediofrontal, and orbitofrontal cortex; (5–9)], brain regions associated with emotion regulation and attention [i.e., parietal cortex, dorsolateral, and dorsomedial cortex; (5, 7, 10)], and for facially expressed social threat also areas in the occipito-temporal and occipital cortex implicated in general face processing (5, 7). Moreover, it was demonstrated recently that SA is also associated with a generally increased cerebral hemodynamic responses to faces and voices as threat carrier signals in the respective sensory face and voice processing areas and the amygdala (11). Taken together, in SA there is evidence for increased hemodynamic cerebral responses to facial and vocal signals of social threat but also to faces and voices in general. As common carrier signals of social threat, faces and voices might be construed as generally more salient communication signals in socially anxious than in socially non-anxious individuals. However, to date it remains unclarified how audiovisual face-voice stimuli as one of the most common forms of natural human social communication are processed and integrated at the cerebral level.

In the present study, we aimed to bridge the astonishing gap between the scientific need for data on the audiovisual processing and integration of nonverbal cues from face and voice in SAD (12) and the complete lack of studies on the neuronal underpinnings of these processes in SAD. The major candidate regions for the occurrence of SAD-related alterations in the audiovisual integration of facial and vocal nonverbal cues are the multisensory temporal cortex adjoining the superior temporal sulcus (STS) and the amygdala based on research in healthy individuals [for reviews see: (13, 14)] and first studies in other psychiatric disorders [e.g., (15, 16), reviewed in (14)].

In 18 individuals with SAD and 18 low SA healthy controls, we investigated how SAD affects the magnitude and topography of cerebral face-voice-integration. The latter was determined using a functional magnetic resonance imaging (fMRI) experiment comparing the responses to dynamic bimodal face-voice combinations displaying various expressions with the monomodal presentations of the stimuli (i.e., only faces or voices). Based on the assumption that voices and faces would be more salient to individuals with SAD than to low socially anxious individuals we hypothesized that in SAD stronger hemodynamic responses to face-voice-combinations would be found in the multisensory integration area of the posterior STS and the amygdala. Second, we expected the increase in hemodynamic activation to occur for face-voice-combinations in comparison to both, faces and voices alone due to the expected increase in stimulus salience compared with the unimodal stimuli.

The standard voxel-by-voxel analysis was complemented with the spatial analysis of individual integration maxima and functional connectivity (FC) analyses to further characterize the hemodynamic correlates of face-voice-integration. Additional fMRI experiments were performed to determine the cerebral voice- and face-sensitivity measured as increased hemodynamic responses to voices or faces, respectively, in comparison to other types of stimuli as additional source of information on the functional characteristics of areas with SAD-related alterations in face-voice-integration.

## Materials and Methods

### Participants

Eighteen individuals with SAD (SAD: six male, mean age 23.3 years, SD 3.3 years) and 18 low socially anxious healthy control individuals (HC: eight male, mean age 24.8 years, SD 2.0) years were included in the study. They were recruited at the Universities of Tübingen (n = 23) and Greifswald (n = 13) through notices in public venues and email circulars at the Universities of Tübingen and Greifswald. All participants underwent the Structured Clinical Interview for DSM-IV [SCID; (17)]. In the participant group with SAD, SAD was the primary diagnosis in all individuals. Three individuals had an additional diagnosis of specific phobia, two had a minor depression, and five had a history of major depressive disorder. In the low socially anxious group, two participants suffered from a specific phobia, one had a minor depression, and two had a history of major depressive disorder. All participants with a history of major depression had been in remission for more than 6 months. SA severity was measured with the Liebowitz Social Anxiety Scale [LSAS; German self-report version, (18)], and general anxiety was assessed using the State-Trait-Anxiety-Inventory [STAI, German version; (19)]. Verbal intelligence was evaluated with the “Mehrfachwahl-Wortschatz-Intelligenz-Test” [MWT-B; (20)]. For a socio-demographic and psychometric overview see **Table 1**. All participants were right-handed according to the Edinburgh Inventory (21), native speakers (German), and reported normal or corrected to normal visual acuity and normal hearing. None of the participants had a history of substance abuse, or neurological illness, or was taking any regular medication. An expense allowance was given for study participation.

**Table 1:** Demographic and psychometric sample information.

	SAD (n=18)	HC (n=18)		
gender	12 f, 6 m	10 f, 8 m	χ^2^ = 0.5	p = 0.49
age	23.3 (3.3)	24.8 (2.0)	t = 1.6	p = 0.11
LSAS	72.2 (22.3)	12.8 (9.7)	t = 10.4	p < 0.001
STAI-X1	43.4 (7.5)	33.6 (7.2)	t = 4.0	p < 0.001
STAI-X2	48.6 (7.5)	37.3 (8.2)	t = 4.3	p < 0.001
MWT-B	32.1 (2.4)	30.7 (2.6)	t = 1.7	p = 0.11
study site	13 T, 5 G	10 T, 8 G	χ^2^ = 1.1	p = 0.30

LSAS, Liebowitz Social Anxiety Scale; STAI, State Trait Anxiety Inventory; MWT-B, “Mehrfachwahl-Wortschatz-Intelligenz-Test”, a short test of premorbid intelligence. Values in parentheses indicate standard deviation of the mean. T, Tübingen, G, Greifswald.

### Ethics Statement

The protocol of human investigation was approved by the local ethics committees where the study was performed (i.e., Universities of Tübingen and Greifswand, Germany), and the study was performed according to the Code of Ethics of the World Medical Association (Declaration of Helsinki, 1964) and its later amendments. All participants gave written informed consent before their inclusion into the study.

### Stimulus Material and Experimental Design

#### Face-Voice-Integration Experiment

The stimulus material consisted of videos (1.6 s) of faces speaking 60 words with neutral or emotional (i.e., angry, disgusted, alluring, fearful, happy, or sad) prosody and congruent facial expression. The stimuli were recorded from six professional actors, allocated to 12 blocks with five stimuli each, and were all presented auditorily (A), visually (V), and audiovisually (AV) totaling in 180 stimuli per participant. Across blocks, the stimuli were randomized on condition that the blocks were balanced for the depicted emotion and the gender of the speaker. Within the blocks, stimulus presentation was randomized. Likewise, within each modality the block sequence was randomized while the modalities of the stimulus blocks were randomized on condition that the block sequence did not contain more than two adjacent blocks from one modality. Auditory stimulation was carried out with MR compatible headphones (MR confon GmbH, Magdeburg, Germany). Visual stimuli were back-projected onto a screen placed in the magnet bore behind the participant's head and viewed by the participant through a mirror system mounted onto the head coil. The participants were asked to indicate the second “male” stimulus within each block by pressing a button on a fiber optic system (LumiTouch, Photon Control, Burnaby, Canada) with their right index finger to ensure constant attention to the stimuli. The experimental design was validated previously and further details on stimulus material and experimental design can be found elsewhere (22).

#### Voice- and Face-Sensitivity Experiments

The voice-sensitivity experiment was designed in form of a block design experiment with 24 stimulations blocks and 12 silent periods (each 8 s) with a passive-listening task and stimulus material based on the study by Belin et al. (23). The stimulus material included 12 blocks of human vocal sounds [e.g., onomatopoeia, speech (e.g., single syllables, finish language), sighs], six blocks of animal sounds (e.g., gallops, various cries), and six blocks of environmental sounds (e.g., cars, planes, doors, telephones). The participants were asked to listen with closed eyes. Stimuli were normalized regarding their mean acoustic energy. Sound and silence blocks were randomized across the experiment.

In the face-sensitivity experiment, pictures from four different categories (faces, houses, objects, and natural scenes) were shown within a block design. The stimulus material and design has been adapted from previous face processing studies (24, 25). Each category and block (duration: 16 s) consisted of 20 pictures. Of each category eight blocks were presented in pseudorandomized order. A one-back task was employed to establish constant attention to the stimuli. The participants were asked to press a button on the fiber optic system (LumiTouch, Photon Control, Burnaby, Canada) whenever a picture was directly repeated.

Both experimental designs have been used previously (22, 26, 27) and further details on stimulus characteristics and design can be found in the respective publications.

### Image Acquisition

MRI data were recorded with a VERIO 3T (Greifswald) and a PRISMA (Tübingen) scanner (Siemens, Erlangen, Germany). Functional images (34(/30) (parenthesized values after the slash refer to the PRISMA scanner throughout this section) axial slices captured in sequential descending order, 3 mm thickness + 1 mm gap, TR = 2.0(/1.7) s, TE = 30 ms, voxel size: 3x3x4 mm^3^, field of view 192x192 mm^2^, 64x64 matrix, flip angle 90°) and structural T1-weighted images [VERIO(/PRISMA): 176 slices, TR = 1900(/2300) ms, TE = 2.52(/2.96) ms, voxel size: 1x1x1 mm^3^] were acquired. The time series comprised 204(/239) images for the face-voice-integration experiment, 302(/368) images for the face experiment and 195(/232) images for the voice experiment. A field map [34(/36) slices, slice thickness 3 mm, TR = 488(/400) ms, TE(1) = 4.92(/5.17) ms, TE(2) = 7.38(/7.65) ms] was recorded.

### Analysis of Demographic and Psychometric Data

IBM SPSS Statistics Version 25 (IBM Corporation, Armonk, NY, USA) was used for the analyses. Differences between SAD and HC with regard to gender, study site (chi square tests), age, SA severity (LSAS), general state and trait anxiety (STAI-X1, STAI-X2), and verbal intelligence (MWT-B) (t tests) were systematically investigated.

### Analysis of Imaging Data

Statistical parametric mapping software (SPM8; http://www.fil.ion.ucl.ac.uk/spm) was employed for imaging data analysis. To exclude measurements preceding T1 equilibrium, the first five EPI images from each fMRI experiment were removed. The preprocessing included realignment, unwarping employing a static field map, coregistration of anatomical and functional images, segmentation of the anatomical images, normalization into MNI (Montreal Neurological Institute) space with a resampled voxel size of 3 × 3 × 3 mm³, temporal smoothing with a high-pass filter (cutoff frequency of 1/128 Hz), and spatial smoothing using a Gaussian kernel (8 mm full width at half maximum). The error term was modeled as an autoregressive process (first order, coefficient: 0.2 with additional white noise component) (28). The realignment parameters (i.e., translation and rotation on the x-, y- and z-axes) were included as covariates in the models at single subject level. For details on the obtained realignment parameters see the Supplemental material (**Table S1**).

The hemodynamic responses to the different stimulus classes [auditory (A), visual (V), and audiovisual stimuli (AV) in the face-voice-integration experiment, voices (V), animal sounds (A), and environmental sounds (E) in the voice-sensitivity experiment, and faces (F), houses (H), objects (O), and scenes (S) in the face-sensitivity experiment] were separately modeled using a box-car function representing the block duration (8 s in the voice-face-integration experiment and the voice-sensitivity experiment and 16 s in the face-sensitivity experiment) convolved with the HRF. Results from the individual first-level general linear models were used to produce contrast images [voice-face-integration: AV - max(A, V); voice-sensitivity: V - max(A, E); face-sensitivity: F – max(H, O, S)] for each participant which were then included in second-level random-effect analyses. These minimum difference contrasts were computed for each participant as the minimum of the contrast images AV – A and AV – V for face-voice-integration, V – A and V – E for voice-sensitivity and F – H, F – O and F – S for face-sensitivity. Age, gender and study site were included as covariates in all second level analyses.

The analytical strategy comprised two arms:

1.) Voxel-wise analysis of the audiovisual integration effect [i.e., AV – max(A, V)] in the whole study sample, in SAD, and in HC as well as of between group differences regarding the audiovisual integration effect (i.e., SAD – HC). Statistical significance was assessed at p < 0.001 at voxel level and cluster-level FWE correction (p < 0.05) for multiple comparisons across the whole brain.
2.) Spatial distribution analysis of the individual audiovisual integration maxima [i.e., position of the maxima for SAD and HC on the three spatial axes and their comparison (t-test)] within the audiovisual integration areas Bonferroni-correcting the results for multiple comparisons across the three spatial axes and two hemispheres (i.e., a total of six comparisons).

Significant effects were post-hoc characterized and validated. To this aim, mean contrast estimates for all three experiments (i.e., face-voice-integration, face-sensitivity, and voice-sensitivity) were extracted from significant clusters. These analyses included the associations of integration effects with parametric measures of social and general anxiety, and individual movement patterns in the scanner (see **Supplemental methods**) as well as the determination of face-sensitivity [i.e., F – max(H,O,S)] and voice-sensitivity [i.e., V – max(A,E)] in the respective areas and potential differences between SAD and HC regarding face- and voice-sensitivity as well as the deconstruction of the integration measure AV – max(A,V) into the underlying basis contrasts AV – A and AV – V including group comparisons for these contrasts and the individual comparison of the two contrast sizes. The latter was done to determine if one of the two basis contrasts was driving the integration effect in the majority of the participants. In the post-hoc analyses, two-tailed testing was applied if not specified otherwise in the *Results* section.

Moreover, the respective clusters were anatomically specified in more detail using the Anatomy toolbox [(29); version 2.2b] integrated in the SPM software.

Apparent discrepancies in SAD-related differences in face-voice-integration effects between voice-sensitive (i.e., the temporal voice area (23) and face-sensitive areas [i.e., the fusiform face area (25) and the face area in the posterior temporal sulcus (30)] as well as the amygdala were post-hoc statistically tested by comparing the audiovisual integration effect AV – max(A,V) between these areas (for details see **Supplemental methods**).

### Psychophysiological Interaction (PPI) Analysis

Finally, we investigated whether areas with SAD-associated alterations in cerebral face-voice-integration also exhibit analogous differences in FC with other brain areas. Areas where SAD differed from HC in the neural correlates of face-voice-integration were defined as seed regions for PPIs. It was tested if changes in FC during audiovisual integration [contrast: AV – max(A, V)] differ between SAD and HC. The BOLD signal time-course of the seed regions, extracted from a sphere (radius: 2 mm) around the individual peak-activation voxel within the seed region adjusted for effects of interest was used as physiological variable. Each condition (i.e., A, V, and AV) was then defined as a separate psychological input variable and contrasted [i.e., AV – max(A, V)]. The PPI was estimated as the product of the vector of the psychological variables and the deconvolved activation time course (31). The physiological and psychological variables and the psychophysiological interaction term were employed as separate regressors in the model. The individual PPI contrasts were submitted to second level random effects analyses (SAD vs. HC) within SPM. Statistical significance was evaluated at p < 0.001 at voxel level and cluster-level FWE correction (p < 0.05) for multiple comparisons across the whole brain. Age, gender and study site were included as covariates in the second level analyses.

## Results

### Sample Characteristics

The psychometric and demographic characteristics of the participant groups (SAD/HC) are given in **Table 1**. SAD differed significantly from HC with regard to SA severity (LSAS) and general anxiety (STAI-X1/X2) but not with regard to gender, age, verbal intelligence (MWT-B) or study site (see also **Table 1**).

### Voxel-Wise Analysis of Face-Voice-Integration

Audiovisual face-voice-integration correlates were observed bilaterally in the temporal cortex adjoining the middle and posterior parts of the STS in the whole sample (see **Table 2** and **Figure S1**). Anatomically, the significant right STS cluster maps partially to the auditory cortex (areas TE 3 >> TE1.2 > TE1.1 > TE 1.0; for details see **Table S2**), but the greater part of the cluster is positioned posterior and inferior to the auditory cortex in the upper bank of the STS.

**Table 2:** Cerebral correlates of face-voice-integration [AV – max(A, V)] in the whole study population.

	Peak coordinate (x y z)	Z-score (peak voxel)	Cluster size (vx)	p value (FWE_corr_)
R superior and middle temporal gyri	54 -33 6	4.8	315	<0.001
L superior temporal gyrus/L Rolandic operculum	-48 -24 12	3.71	66	0.09
L superior and middle temporal gyri	-60 -45 9	3.53	20	0.61

Voxel-wise random effects analysis. Results are shown at a threshold of P < 0.001, uncorrected and a cluster size of >= 10 voxels. Reported p values are FWE-corrected for multiple comparisons across the whole brain. Voxel size: 3 x 3 x 3 mm³. R, right; L, left. Coordinates refer to the MNI system.

**Figure 1:**
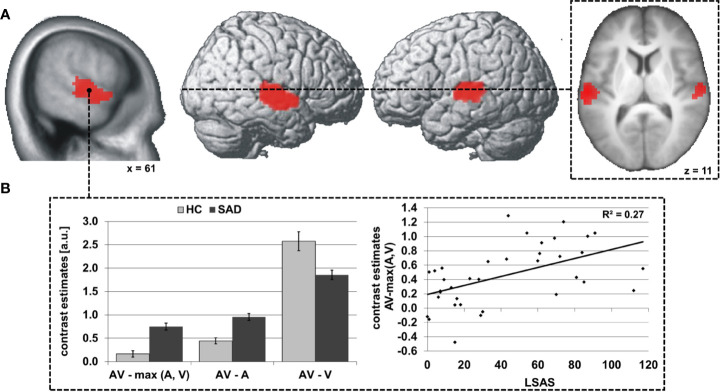
Differences in audiovisual face-voice-integration between SAD and HC rendered onto the lateral aspects of a standard brain as well as sagittal and transversal slices of the whole sample mean anatomy **(A)**. Results shown at p < 0.05, cluster-size FWE-corrected for multiple comparisons across the whole brain at a voxel-wise threshold of p < 0.001. Coordinates refer to the MNI system. Voxel size 3 x 3 x 3 mm^3^. The diagrams **(B)** illustrate the underlying activation differences between audiovisual (AV), auditory (A) and visual (V) stimulation (left) as well as the correlation of the face-voice-integration effect with individual SA severity (LSAS, right). Light columns refer to HC and dark columns to SAD. Error bars indicate the standard error of the mean.

Significant differences between SAD and HC in face-voice-integration were found bilaterally in the superior and middle temporal gyri in the middle part of the STS (see **Table 3** and **Figure 1A**) with stronger audiovisual integration effects for SAD while no area with stronger integration effects for HC was observed. Anatomically, the significant clusters again map partially to the auditory cortex (right STS: areas TE 3 >> TE1.2 >> TE1.0 > TE 1.1; left STS: areas TE 3 >> TE1.2 >> TE1.0; for details see **Table S2**), and again a large part of the cluster is positioned posterior and inferior to the auditory cortex in the upper bank of the STS.

**Table 3:** Group differences (SAD vs. HC) in the cerebral correlates of face-voice-integration [AV – max(A, V)].

SAD > HC	Peak coordinate (x y z)	Z-score (peak voxel)	Cluster size (vx)	p value (FWE_corr_)
R superior and middle temporal gyri/R temporal pole	60 -21 0	4.8	269	<0.001
L superior and middle temporal gyri	-60 -21 6	4.5	186	0.001
L insula	-27 24 3	4.2	37	0.30
L caudate nucleus	-24 -12 30	4.2	46	0.20
L lingual gyrus	-30 -51 0	4.0	22	0.56
R caudate nucleus	18 0 27	3.9	27	0.46
L calcarine cortex/L cuneus	-15 -72 21	3.5	37	0.30
R calcarine cortex/R cuneus	18 -78 6	3.3	52	0.16
**HC > SAD**				
No clusters				

Voxel-wise random effects analysis. Results are shown at a threshold of P < 0.001, uncorrected and a cluster size of >= 10 voxels. Reported p values are FWE-corrected for multiple comparisons across the whole brain Voxel size: 3 x 3 x 3 mm³. R, right; L, left. Coordinates refer to the MNI system.

Further analyses indicated that the audiovisual integration effect AV > max(A,V) was determined by the smaller difference between AV and A stimulation than between AV and V in almost all participants (right STS: 17/18 HC and 18/18 SAD, left STS: 18/18 HC and 18/18 SAD). Additionally, the increased face-voice-integration effect in SAD was statistically driven by stronger increases for AV compared to A in SAD as compared to HC [right/left STS: t(34) = 5.6/5.0, both p < 0.001] while the activation increase under bimodal stimulation as compared to unimodal visual stimulation was decreased in SAD [right/left STS: t(34) = 3.2/3.6, both p ≤ 0.003] (see **Figure 1B**, left). The size of the face-voice-integration effect was linearly associated with individual SA severity (right/left STS: r= 0.52/0.50, both p ≤ 0.002) (see **Figure 1B**, right). The right as well as the left STS area exhibited significant voice-sensitivity [both t(35) ≥ 8.7, both p < 0.001, one-tailed] with no significant difference between SAD and HC {both abs[t(34)] ≤ 0.8, both p > 0.05}. In contrast, the bilateral STS areas were not face-sensitive [both t(35) ≤ -3.6, both p > 0.05, one-tailed], again without group difference {SAD vs. HC, both abs[t(34)] ≤ 0.2, both p > 0.05}.

### Spatial Analysis of Individual Face-Voice-Integration Maxima

**Figure 2:**
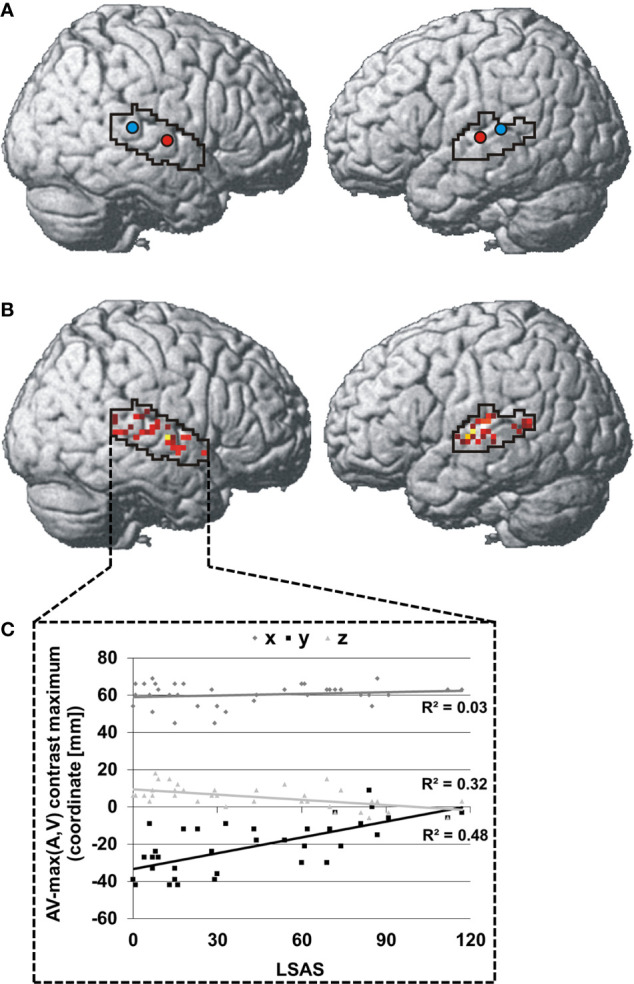
Spatial distribution of individual face-voice-integration maxima: Group centroids (SAD = red and HC = blue) **(A)** and individual face-voice-integration maxima **(B)** rendered onto the lateral aspects of a standard brain. Individual maxima are color-coded in a red-yellow-white spectrum with dark red indicating very low SA severity and white indicating very high SA severity. **(C)** Association between SA severity and the position of the individual face-voice-integration maximum within the right STS shown separately for the three spatial dimensions. Coordinates refer to the MNI system.

The centroid of integration maxima was found significantly more anterior and inferior in SAD than HC in the right STS [SAD: 61.3x -12.0y 2.7z, HC: 59.3x -30.5y 8.3z; x: t(34) = 1.0, p > 0.05; y: t(34) = 5.3, p < 0.001; z: t(34) = 3.3, p = 0.011] and more anterior in the left STS [SAD: -58.3x -18.5y 6.8z, HC: -57.3x -29.3y 7.5z; x: t(34) =0.6, p > 0.05; y: t(34) = 3.0, p = 0.029; z: t(34) = 0.4, p > 0.05] (see **Figure 2A**). The individual locations of the face-voice-integration maxima were linearly related to SA severity on the y-axis and on the z-axis in the right STS (y-axis: r = 0.70, p < 0.001; z-axis: r = 0.56, p = 0.002) (see **Figures 2B, C**).

### Psycho-Physiological Interaction (PPI) Analysis

**Figure 3:**
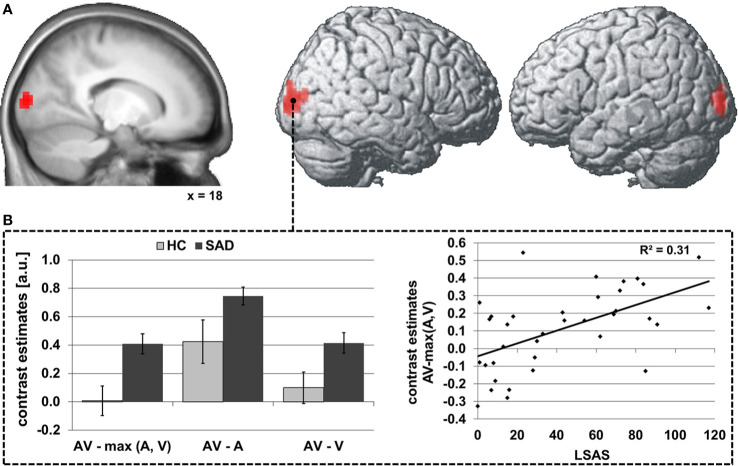
Differences in audiovisual face-voice-integration-related modulations of right STS FC between SAD and HC rendered onto the lateral aspects of a standard brain as well as a sagittal slice of the whole sample mean anatomy (psychophysiological interaction analysis; seed region as depicted in **Figure 1**) **(A)**. Results shown at p < 0.05, cluster-size FWE-corrected for multiple comparisons across the whole brain at a voxel-wise threshold of p < 0.001. Coordinates refer to the MNI system. Voxel size 3 x 3 x 3 mm^3^. The diagrams **(B)** illustrate the underlying differences in FC between audiovisual (AV), auditory (A) and visual (V) stimulation (left) as well as the correlation of the face-voice-integration effect with individual SA severity (LSAS, right). Light columns refer to HC and dark columns to SAD. Error bars indicate the standard error of the mean.

During audiovisual stimulation the right STS exhibited greater FC with the striate and superior peristriate visual cortex in the cuneus and superior occipital gyrus (see **Table 4** and **Figure 3A**) while no significant modulations of FC during face-voice-integration were observed for the left STS (see **Table 4**). Anatomically, the significant occipital cluster corresponds largely to area hOc1 (right > left) with small parts mapping to areas hOc2, hOc3v/d and hOc4d (for details see **Table S2**).

**Table 4:** Effect of SAD on face-voice-integration-related changes [AV – max(A, V)] in functional connectivity (PPI).

Seed: right STSSAD > HC	Peak coordinate (x y z)	Z-score (peak voxel)	Cluster size (vx)	p value (FWE_corr_)
L middle frontal gyrus pars orbitalis	-24 33 -3	4.7	12	0.80
R + L calcarine cortex/R cuneus/R superior occipital gyrus	18 -90 9	4.3	136	0.013
R Middle and superior temporal gyrus	39 -51 6	4.1	78	0.08
R + L caudate nucleus	6 9 9	3.8	43	0.28
**HC > SAD**				
No clusters				
**Seed: left STS**				
**SAD > HC**				
L angular gyrus	-48 -75 -30	3.8	12	0.8
R putamen/R globus pallidus	24 0 -6	3.6	12	0.8
**HC > SAD**				
No clusters				

Voxel-wise random effects analysis. Results are shown at a threshold of P < 0.001, uncorrected and a cluster size of >= 10 voxels. Reported p values are FWE-corrected for multiple comparisons across the whole brain Voxel size: 3 x 3 x 3 mm³. R, right; L, left. Coordinates refer to the MNI system.

The group effect was driven by greater increases in FC during audiovisual stimulation as compared to visual and to auditory stimulation [both t(34) ≥ 2.1, both p ≤ 0.046]. The FC increase during face-voice-integration {i.e., AV-max(A,V) was significant in SAD [t(17) = 6.4, p < 0.001] but not in HC [t(17) = 0.2, p > 0.05]}. The latter lack of a face-voice-integration effect in HC was mainly based on a non-signicant increase in FC during audiovisual than during visual stimulation [t(17) = 2.0, p > 0.05] whereas the FC increase for audiovisual compared to auditory stimulation was significant [t(17) = 3.7, p = 0.002] (see **Figure 1B**, left). The face-voice-integration effect was positively correlated with individual SA severity (r= 0.56, p < 0.001) (see **Figure 3B**, right).

### Validation and Post-Hoc Analyses

Most observed effects remained significant when general anxiety or the individual movement parameters for the integration contrast AV – max(A,V) were included as covariates with the only exception of the relationship between group and the position of the individual integration maximum on the z-axis in the right STS when correcting for general trait anxiety. Moreover, when the positions of the face-voice-integration maxima were included as covariates, the hemodynamic face-voice-integration effect remained significantly associated with the diagnosis of SAD in the bilateral STS clusters (for details see **Supplemental Results**).

Post-hoc follow-up analyses subsequent to the finding of increased hemodynamic correlates of face-voice-integration in SAD in the bilateral voice-sensitive midsection of the STS confirmed that bilaterally the voice-sensitive temporal voice area exhibited increased hemodynamic correlates of face-voice-integration in SAD. Comparable effects were not observed in the amygdalae or the canonical temporal face-sensitive cortex areas (i.e., the fusiform face area and the posterior STS face area). Moreover, the SAD-related increases in face-voice-integration effects in the bilateral temporal voice areas were found to be statistically stronger than in the amygdalae and the face-sensitive cortex areas (for details see **Supplemental Results**).

## Discussion

In the present study, we were able to demonstrate for the first time that both, the intensity and also the topography of the audiovisual integration of facial and vocal cues at the cerebral level are specifically related to SAD irrespective of concomitantly elevated levels of general anxiety. The response patterns in the bilateral temporal cortex adjacent to the midsection of the STS indicate that the increased face-voice-integration effect in SAD is driven by a greater response increase for audiovisual as compared to auditory stimuli. Moreover, based on the location and response profiles with voice- but not face-sensitivity, this area appears rather to be a part of the so-called temporal voice area [TVA; (23, 32)] than the multimodal cortex of the pSTS which additionally exhibits face-sensitivity (33). Thus, in contrast to our *a priori* assumption, SAD-related face-voice-integration effects occurred in a modality-selective rather than a multimodal cortex area and were driven selectively by a greater response increase in SAD through the addition of faces to voices in this area while no comparable effect could be observed for the comparison of face-voice-combinations and faces alone in this area. Potentially, the combined perception of congruent signals from voices and faces in contrast to voices alone, specifically boosts this area's activation in SAD as an indicator of increased salience of multimodal face-voice-combinations in comparison to voices alone. This observation fits in well with and extends the findings of previous studies with meta-analytic evidence for assumedly salience-related increased hemodynamic responses in the STS during unimodal face processing [e.g. (34)]. Further, our results extend findings from a recent fMRI study demonstrating that increased hemodynamic responses in SAD occur within the STS not only for unimodal faces in the face-sensitive area of the pSTS but also for unimodal voices in the TVA (11).

Moreover, as the site of increased face-voice-integration correlates corresponds well with the posterior maximum of the TVA slightly medial of area TE3 in the temporal cortex (32), increased extraction of meaningful patterns from the voice (35, 36) in the context of congruent face cues in SAD may provide an explanation for the activation increase. And as there additionally is a striking spatial convergence with the emotional voice area (37), “meaningful” might well be equivalent with “emotional” in this instance. Then again, keeping in mind that nonverbal emotional signals are often perceived as threatening in SAD, the observed phenomenon might be related to increased extraction of signs of social threat from voices in the context of faces. Further, the increased FC with the striate and upper peristriate visual cortex may support this process potentially indicating a contribution of visual cue characteristics to the auditory information extraction. Dovetailing with this interpretation, corresponding connectivity effects indicating an auditory contribution to the processing of visual cues have been reported (38, 39) suggesting a contribution of such crossmodal effects even between cortical areas assumed to be unimodal association areas during the processing of multimodal stimuli. Interestingly, the connectivity effect did not occur in a face-sensitive area but in a region which suggests that lower level visual stimulus characteristics and not a holistic face representation inform the increased extraction of auditory information.

Also, SAD-related increased hemodynamic activation during audiovisual integration the voice-sensitive auditory association cortex was statistically stronger than within the corresponding face-sensitive cortices of the pSTS (40) and the fusiform gyrus (41) where no comparable effects could be observed. And without the aim of overinterpreting this response difference between voice- and face-sensitive cortex areas, one may cautiously speculate on the potential reason for this discrepancy. One explanation might be found in the visual bias in emotion perception which manifests in a greater ability to identify emotional information from the face component than the voice component of audiovisual nonverbal expressions [e.g., (42, 43)]. Therefore, one may assume that for the contrast AV – A there is a greater gain in perceived nonverbal information than for the contrast AV – V. Now, if this visual bias was increased in SAD, this might potentially explain the observed discrepancies between voice-sensitive or face-sensitive cortex areas. Additionally, it could be speculated that a comparable SAD-related integration effect in face-sensitive cortices might arise when the perceptual gain between AV and V is experimentally increased (e.g., through visual stimulus degradation).

Interestingly, this putative mechanism might also explain that SAD-related activation increases during face-voice-integration in SAD in the amygdalae were statistically weaker than in the voice-sensitive midsection of the STS and did not reach any level of significance. The amygdala has been discussed to be central in the integration of stimulus salience across different nonverbal communication channels (44). It could be argued that the visual bias in the recognition of facial and vocal expressions might also reflect greater stimulus salience of facial as compared to vocal expressions. Accordingly, the considerable increase in expression recognition rates for face-voice-combinations as compared to voices and the much smaller increase for the comparison with faces [e.g., (42, 43)] might correspond to equivalent increases in stimulus salience. Fittingly, this hypothetical impact of a potentially also SAD-related visual bias on the processing of naturalistic nonverbal emotional signals could be tested with the same experimental approach as described above.

The significant linear associations between the hemodynamic and functional connectivity correlates of face-voice-integration in the STS and SA severity suggest that they reflect spectral cerebral features of SA in the population and are not necessarily bound to the clinical significance of SA with a diagnosis of SAD.

The topographical analysis of the individual integration maxima, unexpectedly, revealed an anteriorization of the maximal integration effects along the STS irrespective of individual movement patterns in the scanner as a second component of the cerebral representation of face-voice-integration in SAD. Validation analyses confirmed the complimentary nature of the effects observed in the two analyses focused on the magnitude and the location of face-voice-integration effects. In other words, irrespective of the location of the individual face-voice-integration maximum, SAD is associated with increased hemodynamic face-voice-integration correlates. Thus, the inclusion of this non-standard type of data analysis in future studies may afford an additional informative perspective on the cerebral representation of SAD but also other psychiatric disorders and, keeping in mind the spectral nature of the effect, potentially also individual psychometric characteristics.

And also from the point of view of sensory integration research, these results might be relevant. It appears that the identical mathematical measure of integration could reflect different underlying neuronal processes: the integration of vocal and facial expressions into a unified percept in the multisensory neurons of the pSTS (14, 42) in healthy individuals as initially framed concept based on electrophysiological studies, but also the increased extraction of socially relevant information from voices in the context of congruent facial cues in the TVA depending on interindividual differences in SA. If this was the case, it would be advisable to closely monitor SA and potentially other individual psychometric characteristics as covariates in this area of research.

From the clinical perspective, our results suggest that SAD-related cerebral alterations in social cognition exceed the domains of unimodal social cue processing as previously demonstrated for faces (11, 34) and voices (11), and also encompass the process of multimodal integration of these signals. This finding not only corroborates the assumption of a broader alteration in social cognition (34) but may also be helpful for a deeper understanding of the cerebral underpinnings of SAD pathogenesis and might bear implications of novel therapeutic approaches.

### Limitations and Perspectives

The present design established a clear link between SAD and the increased hemodynamic integration correlates of vocal and facial information. However, the functional relevance of the observed activation patterns remains unclear. Therefore, further studies including measures of SAD-related behaviors (e.g., cognitive biases) are needed to elucidate if the observed effects potentially reflect a consequence of dysfunctional perceptive learning, either as correlates underpinning pathological processes in convergence with current models of SA (4, 45), or rather compensatory mechanisms against cognitive biases through audiovisual integration of redundant social information. Then again, the opposite might be true, and the cerebral alterations could reflect a genetically determined factor in voice and face processing (46, 47) as a predisposition for the development of severe SA.

Including dynamic displays and several different types of nonverbal expressions, the stimulus material was composed to encompass a broad range of naturally occurring nonverbal social communication. However, the study design does not allow the differentiation between neutral and emotionally negatively or positively valenced expressions all of which are subject to negative cognitive biases (10, 48–52) but may be experienced as socially threatening to a different degree. Therefore, more research is needed to evaluate these factors with regard to their influence on the observed effects.

Finally, two further points should be noted: First, the sample size in our study was relatively small. Second, correction for multiple comparisons based on cluster size in combination with standard voxel-wise statistical thresholds in the SPM software has been criticized as potentially too lenient with an increased risk of false positives, especially in studies with weakly significant results (53, 54). Therefore, a replication of the results in future studies would be desirable to rule out the albeit relatively small risk of false positives in the present study and obtain a more exact estimate of the true effect sizes.

## Conclusion

In summary, our study connected two important areas of neuroscience: the research on threat-related signal processing in SAD and the research on the sensory and perceptual integration of signals from face and voice. We demonstrated increased hemodynamic face-voice-integration correlates in SAD anterior to the multisensory cortex of the pSTS within the TVA and a concomitant increase in FC with the striate and peristriate visual cortex. It appears plausible that these effects reflect increased extraction of emotional information from voices supported by an enhanced contribution of visual cue characteristics to voice processing potentially also mirroring the neural processes underlying increased salience of bimodal face-voice-combinations as compared to voices alone in SAD. Further, our results show not only an increase in the hemodynamic correlates of face-voice-integration but also SAD-related crossmodal interactions in voice-sensitive areas (i.e., the TVA) during the perception of naturalistic dynamic face-voice-combinations. These are potentially indicative of a shift of the intermodal balance between voice and face processing during the perception of audiovisual nonverbal signals. Validation in future studies provided, it is quite conceivable to investigate the clinical and therapeutic relevance of this intermodal imbalance for example in form of a perceptual training study aiming to rebalance the processing of faces and voices. Moreover, the topographic analysis highlighted the anteriorization of the individual integration maxima as an additional component of the cerebral representation of SAD and advocates this analysis type also for studies in other psychiatric disorders and individual psychometric characteristics. Still, there remains the evident need for further research to fully unravel the neural bases of the multimodal integration of social threat and that of social threat carrier signals like faces and voices in SA.

## Data Availability Statement

The datasets generated for this study are available on request to the corresponding author.

## Ethics Statement

The studies involving human participants were reviewed and approved by Ethik-Kommission an der Medizinischen Fakultät der Eberhard-Karls-Universität und am Universitätsklinikum Tübingen. The patients/participants provided their written informed consent to participate in this study.

## Author Contributions

BK, DW, ME, and CB conceived and designed the study. SW, ML, TE, ME, CB, and BK acquired the data, and BK, DW, CB, TE, SW, and AW analyzed and interpreted the data. BK drafted the manuscript and all authors critically revised the manuscript.

## Funding

This work was supported by grants of the Fortüne-Program of the University of Tübingen (fortüne 1997-0-0, and fortüne 2140-0-0) and the German Research Foundation Open Access Publication Fund.

## Conflict of Interest

The authors declare that the research was conducted in the absence of any commercial or financial relationships that could be construed as a potential conflict of interest.
