# Supplementary material for: The Neural Correlates of Face-Voice-Integration in Social Anxiety Disorder
Source: Front Psychiatry. 2020 Jul 15;11:657. doi: 10.3389/fpsyt.2020.00657 (PMC7381153; doi:10.3389/fpsyt.2020.00657)
Supplement: Supplementary file 1 [file DataSheet_1.doc]

**Supplemental material**

**Supplemental methods**

**Post-hoc characterization and validation analyses**

Post-hoc characterization included the decomposition of the audiovisual integration effect and the investigation of associations with SA severity (LSAS). Validation analyses comprised the replication of the initial analyses regarding group differences and LSAS-associations with general anxiety estimates (STAI) included as covariates in partial correlations to ensure that observed effects were reliably related to social aspects of anxiety.

In cases where the spatial pattern of audiovisual integration maxima (i.e. x-, y-, and/or z-coordinates) were associated with group (SAD vs. HC)/ LSAS scores, validation analyses were run to exclude that the effect was due to general anxiety or group-/ LSAS-associated movement differences in the scanner despite the inclusion of the realignment parameters as covariates in the first level models. To this end, STAI scores and the individual average movement extent contrast calculated analogously to the audiovisual integration criterion AV – max(A, V) from the realignment parameters were tested for significant associations with group/ LSAS-scores and included as covariates in partial correlations retesting the association between the individual location of the audiovisual integration maximum and group/ LSAS scores.

The final set of validation analyses included the investigation of potential associations between group differences in audiovisual integration in the voxel-wise analysis and the location of the individual audiovisual integration maxima within the respective cluster: The coordinates of the audiovisual integration maxima within the group difference clusters were tested for group differences /associations with LSAS scores and associations with mean audiovisual integration effect from the respective cluster. Those coordinates of audiovisual integration maxima associated with group/ LSAS scores were used as covariates in partial correlations between the cluster’s mean contrast estimates and group/ LSAS scores to exclude that observed effects were solely due to the location of the integration maximum within the respective cluster.

**Post-hoc comparison of SAD-related differences in face-voice-integration effects between voice-sensitive and face-sensitive cortex areas as well as the amygdala**

The observed increase in hemodynamic correlates of face-voice-integration in SAD in the voice-sensitive midsection of the bilateral STS and the absence of comparable effects in canonical face-sensitive areas (i.e., the fusiform face area, FFA, [1](#_ENREF_1), and the posterior STS face area, pSTSFA, [2](#_ENREF_2)) and the amygdala was analyzed in more detail. Specifically, it was investigated if the voice-sensitive cortex in the STS (i.e., the temporal voice areas, TVA, ) exhibit statistically stronger hemodynamic correlates of face-voice integration than the face-sensitive cortex areas (i.e., the pSTSFA and the FFA) and the amygdalae. To this aim, anatomical and functional ROIs were defined. The Anatomy toolbox ([5](#_ENREF_5); version 2.2b) integrated in the SPM software was used for the definition of the left and right amygdala ROIs (size: left amygdala: 102 voxels (voxel size: 3 x 3 x 3 mm³), right amygdala: 93 voxels). The size of the functional ROIs was defined to match that of the anatomical ROIs as closely as possible. Therefore, the most voice-sensitive 97 voxels (approximating the average size of the amygdala ROIs) in the right and left STS were defined as the right TVA and the left TVA, respectively, based on the minimum difference statistic V – max(A,E) (right: Z_max_ = 6.91 at 54x -33y 0z; left: Z_max_ = 7.45 at -60x -9y -3z) while the most face-sensitive voxels in the right STS were defined as the right pSTSFA based on the minimum difference statistic F – max(H,O,S) (Z_max_ = 5.03 at 57x -63y 9z). In the case of the right FFA only 28 voxels exhibiting face-sensitivity above a voxel-wise threshold of p < 0.05, uncorrected, could be localized (Z_max_ = 3.33 at 42x -45y -18z) and were defined as the right FFA. In the left hemisphere no face-sensitive voxels above a voxelwise threshold of p < 0.05, uncorrected, could be localized using the minimum difference statistic F – max(H,O,S). Thus, in the present sample, no left FFA and no left pSTSFA could be determined based on the minimum difference statistic F – max(H,O,S). Mean contrast estimates for AV – max(A,V) were extracted from the ROIs. Group differences in face-voice-integration were calculated using two-sample t-tests, and comparisons were performed between the right and the left TVA and every other ROI, respectively. This was done applying two series of four 2 x 2 repeated measures ANOVAS with ROI as within-subject factor and participant group (SAD, HC) as between subject factor. The interaction term was evaluated as measure of interest. For the two-sample t-tests, the results were Bonferroni-corrected for the number of ROIs (i.e., six), and for the ANOVAs, they were Bonferroni-corrected for the number of comparisons within each series of ANOVAs (i.e., four). Effect sizes (Cohen’s d) including the respective 95% confidence intervals were calculated using SPSS software (IBM Corp., released 2019, IBM SPSS Statistics for Windows, Version 26.0, Armonk, NY) with code by Karl L. Wuensch (Wuensch, K. L. (unknown). CI-d-SPSS.zip incl. data, syntax and instruction files retrieved from http://core.ecu.edu/psyc/wuenschk/SPSS/CI-d-SPSS.zip on the 10th of June, 2020).

**Supplemental results**

**Validation analyses**

General state and trait anxiety was significantly correlated with the individual hemodynamic face-voice-integration effect bilaterally in the STS clusters (right: STAI-X1: r = 0.42, p = 0.012, STAI-X2: r = 0.40, p = 0.014; left: STAI-X1: r = 0.42, p = 0.010, STAI-X1: r = 0.44, p = 0.007), with the position of the individual face-voice-integration maximum in the right but not the left temporal cortex (right: y-axis: STAI-X1: r = 0.50, p = 0.002, STAI-X2: r = 0.74, p < 0.001; z-axis: STAI-X1: r = 0.40, p = 0.014; STAI-X2: r = 0.53, p = 0.001; left: y-axis: STAI-X1: r = 0.25, p > 0.05, STAI-X2: r = 0.26, p > 0.05), and with the face-voice-integration-related modulations of FC between the right STS and the calcarine visual cortex (STAI-X1: r = 0.44, p = 0.008, STAI-X2: r = 0.37, p = 0.027). In all cases of significant associations with general state and trait anxiety, the results for SAD and individual SA severity remained significant when measures of general state and trait anxiety were included as covariates (all r ≥ 0.33, all p ≤ 0.026, one-tailed) with the sole exception of the association between group and the position of the individual integration maximum on the z-axis in the right STS when correcting for general trait anxiety (r = 0.26, p = 0.063, one-tailed).

No significant difference between SAD and HC was observed for any of the participants’ movement parameters for any of the experimental conditions or for the contrast AV – max(A,V) (all t(34) ≤ 1.8, all p > 0.05), nor was there a linear relationship with individual SA severity (all r ≤ 0.33, all p > 0.05). When the individual movement parameters for the integration contrast AV – max(A,V) were included as covariates, all detected associations between SAD/ individual SA severity and the position of the face-voice-integration maximum on the y- and z-axes remained significant (SAD: all r ≥ 0.45, all p ≤ 0.003, one-tailed; SA severity: all r ≥ 0.32, all p ≤ 0.032, one-tailed).

Finally, when the positions of the face-voice-integration maxima on the y- and z-axes in the right STS cluster and on the y-axis in the left STS cluster were included as covariates, the face-voice-integration effect remained significantly associated with the diagnosis of SAD (right STS: all r ≥ 0.71, all p < 0.001; left STS: r = 0.60, p < 0.001) as well as with individual SA severity (right STS: all r ≥ 0.50, all p = 0.001, one-tailed ; lSTS: r = 0.49, p = 0.001, one-tailed).

**Post-hoc comparison of SAD-related differences in face-voice-integration effects between voice-sensitive and face-sensitive cortex areas as well as the amygdala**

In the right and the left TVA stronger hemodynamic correlates of face-voice-integration in SAD as compared to HC were observed (right: t(34) = 3.7, p = 0.005, d = 1.11 (0.40 – 1.81 95%-CI); left: t(34) = 4.6, p < 0.001, d = 1.22 (0.50 – 1.93)) while no between-group differences were found in any of the other ROIs (i.e., amygdala (right: t(34) = -0.2, p > 0.05, d = -0.07 (-0.72 – 0.58); left: t(34) = -0.6, p > 0.05, d = -0.20 (-0.85 – 0.46)), right FFA: t(34) = 0.6 p > 0.05, d = 0.08 (-0.47 – 0.84), and right pSTSFA: t(34) = -1.8, p > 0.05, d = -0.59 (-1.262 – 0.08)).

The increases in SAD in the right and in the left TVA were stronger than in any of the other ROIs (right TVA comparisons: amygdala: right: F(1,34) = 11.2, p = 0.008, d = 1.11 (0.40 – 1.81), left: F(1,34) = 13.4, p = 0.004, d = 1.22 (0.50 – 1.93); right FFA: F(1,34) = 7.4, p = 0.04, d = 0.91 (0.21 – 1.59); right pSTSFA: F(1,34) = 25.1, p < 0.001, d = 1.67 (0.90 – 2.42); left TVA comparisons: amygdala: right: F(1,34) = 20.1, p < 0.001, d = 1.50 (0.74 – 2.23), left: F(1,34) = 20.7, p < 0.001, d = 1.22 (0.76 – 2.25); right FFA: F(1,34) = 16.4, p = 0.001, d = 1.35 (0.62 – 2.07); right pSTSFA: F(1,34) = 33.3, p < 0.001, d = 1.92 (1.12 – 2.71).

**Supplemental tables:**

Table S1 Realignment parameters obtained from the study sample:

|  | mean | SD | min | max |
| --- | --- | --- | --- | --- |
| translation (mm) |  |  |  |  |
| x-axis |  |  |  |  |
| average scan to scan | 0.0168 | 0.0095 | 0.0046 | 0.0469 |
| maximum scan to scan | 0.1227 | 0.1110 | 0.0283 | 0.5260 |
| maximum across scans | 0.2673 | 0.1484 | 0.0595 | 0.6437 |
| y-axis |  |  |  |  |
| average scan to scan | 0.0262 | 0.0130 | 0.0082 | 0.0597 |
| maximum scan to scan | 0.1787 | 0.1418 | 0.0319 | 0.7356 |
| maximum across scans | 0.2780 | 0.2184 | 0.0738 | 1.1710 |
| z-axis |  |  |  |  |
| average scan to scan | 0.0387 | 0.0209 | 0.0165 | 0.1059 |
| maximum scan to scan | 0.3967 | 0.3876 | 0.0767 | 1.5949 |
| maximum across scans | 0.7892 | 0.7019 | 0.1337 | 3.0281 |
| rotation (degrees) |  |  |  |  |
| x-axis |  |  |  |  |
| average scan to scan | 0.0377 | 0.0169 | 0.0207 | 0.0979 |
| maximum scan to scan | 0.3978 | 0.4437 | 0.0668 | 2.0022 |
| maximum across scans | 0.7845 | 0.7481 | 0.0934 | 3.6993 |
| y-axis |  |  |  |  |
| average scan to scan | 0.0169 | 0.0076 | 0.0084 | 0.0414 |
| maximum scan to scan | 0.1512 | 0.2629 | 0.0305 | 1.6161 |
| maximum across scans | 0.3548 | 0.3222 | 0.0815 | 1.3438 |
| z-axis |  |  |  |  |
| average scan to scan | 0.0165 | 0.0073 | 0.0059 | 0.0351 |
| maximum scan to scan | 0.1449 | 0.1517 | 0.0365 | 0.7392 |
| maximum across scans | 0.3946 | 0.2728 | 0.0700 | 1.2512 |

SD = standard deviation

**Table S2** Anatomical specification of areas with significant face-voice-integration-related changes (AV – max(A, V)) in hemodynamic responses and functional connectivity (PPI)

|  | Anatomical specification |
| --- | --- |
| Hemodynamic responses |  |
| Whole sample |  |
| Right STS cluster | 27.7% of the cluster volume are assigned in total |
|  | 19.1 % area TE 3 |
|  | 2.6 % area TE 1.2 |
|  | 1.4 % area Id1 |
|  | 1.4 % area TE 1.1 |
|  | 1.1 % area Ig2 |
|  | 0.8 % area TE 1.0 |
|  | 0.7 % area OP1 [SII] |
|  | 0.5 % area OP4 [PV] |
|  | 0.1% area Ig1 |
| Group differences: SAD > HC |  |
| Right STS cluster | 51.5 % of the cluster volume are assigned in total |
|  | 42.6 % area TE 3 |
|  | 7.4 % area TE 1.2 |
|  | 1.3 % area TE 1.0 |
|  | 0.1 % area TE 1.1 |
|  | 0.1% area OP4 [PV] |
| Left STS cluster | 43.5% of the cluster volume are assigned in total |
|  | 31.7 % area TE 3 |
|  | 8.4 % area TE 1.2 |
|  | 3.0 % area TE 1.0 |
|  | 0.2 % area Id1 |
|  | 0.2 % area OP4 [PV] |
| PPI group differences: SAD > HC |  |
| Occipital cluster | 87.0 % of the cluster volume are assigned in total |
|  | 63.5 % right area hOc1 [V1] |
|  | 21.1 % left area hOc1 [V1] |
|  | 1.9 % right area hOc3v [V3v] |
|  | 0.2 % right area hOc3d [V3d] |
|  | 0.1 % right area hOc2 [V2] |
|  | 0.1 % left area hOc2 [V2] |
|  | 0.1% right area hOc4d [V3A] |

Values as extracted from the Anatomy toolbox (version 2.2b) integrated in the SPM software.

**Supplementary references:**

1. N. Kanwisher, J. McDermott and M. M. Chun: The fusiform face area: a module in human extrastriate cortex specialized for face perception. *J Neurosci*, 17(11), 4302-11 (1997)

2. J. V. Haxby, E. A. Hoffman and M. I. Gobbini: The distributed human neural system for face perception. *Trends Cogn Sci*, 4(6), 223-233 (2000)

3. P. Belin, R. J. Zatorre, P. Lafaille, P. Ahad and B. Pike: Voice-selective areas in human auditory cortex. *Nature*, 403(6767), 309-12 (2000)

4. C. R. Pernet, P. McAleer, M. Latinus, K. J. Gorgolewski, I. Charest, P. E. Bestelmeyer, R. H. Watson, D. Fleming, F. Crabbe, M. Valdes-Sosa and P. Belin: The human voice areas: Spatial organization and inter-individual variability in temporal and extra-temporal cortices. *Neuroimage*, 119, 164-74 (2015)

5. S. B. Eickhoff, K. E. Stephan, H. Mohlberg, C. Grefkes, G. R. Fink, K. Amunts and K. Zilles: A new SPM toolbox for combining probabilistic cytoarchitectonic maps and functional imaging data. *Neuroimage*, 25(4), 1325-35 (2005) doi:10.1016/j.neuroimage.2004.12.034
