# Supplementary figures and images for: The Neural Correlates of Face-Voice-Integration in Social Anxiety Disorder
Source: Front Psychiatry. 2020 Jul 15;11:657. doi: 10.3389/fpsyt.2020.00657 (PMC7381153; doi:10.3389/fpsyt.2020.00657)

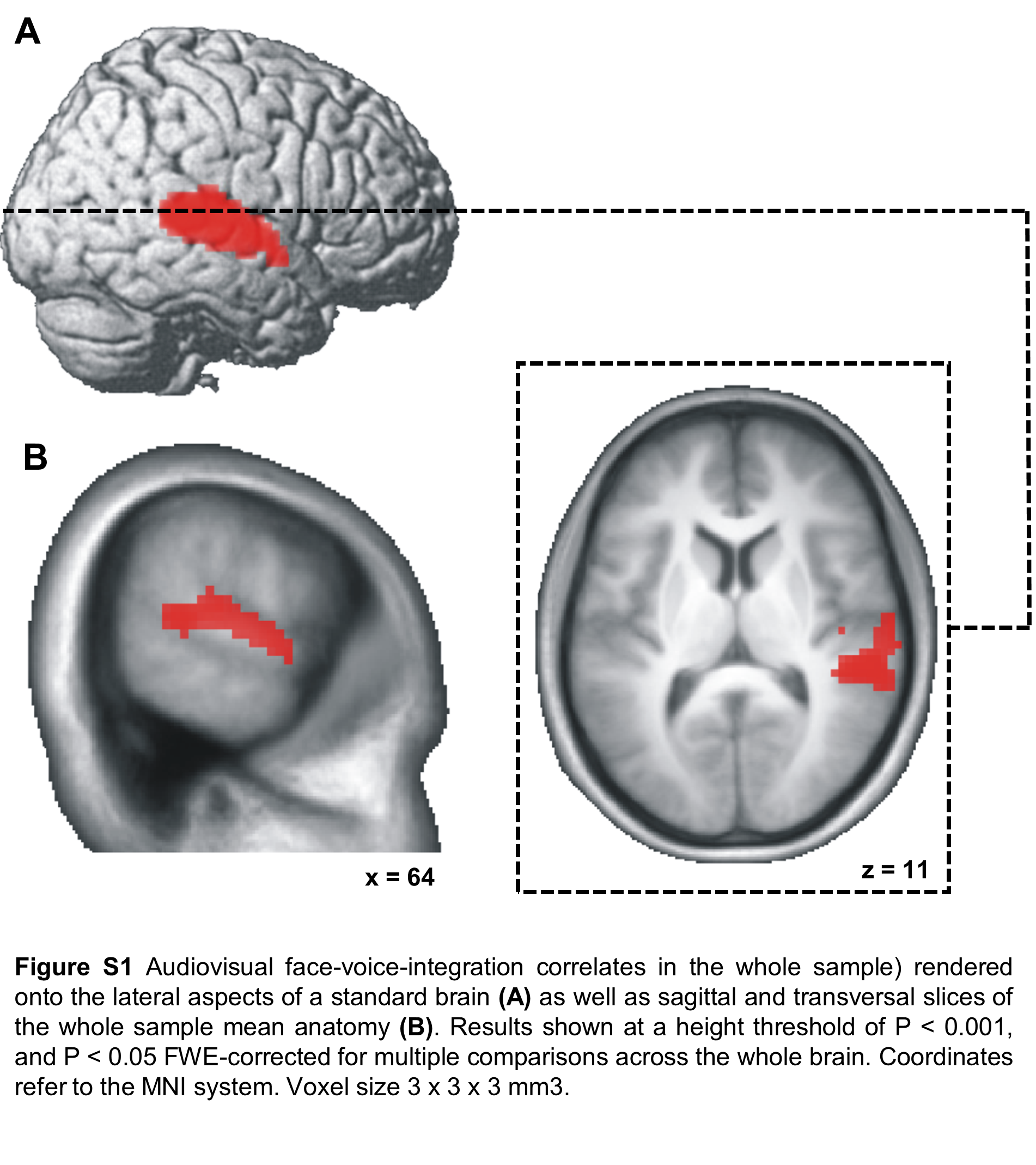

Supplement: Supplementary file 2 [file Image_1.tif]
